# A narrative review of challenges faced by informal caregivers of people with dementia in the Middle East and North Africa

**DOI:** 10.3389/fmed.2025.1610957

**Published:** 2025-09-11

**Authors:** Maha Al-Namla, Anns Mahboob, Eman Radwan, Zena Sinan, Abdulrahman Al-Namla, Mohamud A. Verjee, Ali Chaari

**Affiliations:** ^1^Weill Cornell Medicine–Qatar, Qatar Foundation, Education City, Doha, Qatar; ^2^Cumming School of Medicine, University of Calgary, Calgary, AB, Canada

**Keywords:** caregiver burden, culture, dementia, economy, informal caregiving, MENA, quality of life

## Abstract

**Background:**

Informal caregivers of individuals with dementia in the Middle East and North Africa (MENA) region face a unique set of challenges shaped by cultural, religious, and structural factors. Understanding these challenges is crucial for informing effective supportive interventions.

**Methods:**

A narrative review was conducted by searching PubMed, Scopus, Medline, Embase, and Web of Science in January 2024. Thirty-two studies that met the inclusion criteria were analyzed using an inductive thematic approach to synthesize findings related to caregiver burden across the MENA region.

**Results:**

Key themes identified include financial strain, gendered burden, inadequate governmental support, limited dementia knowledge, and reliance on domestic workers. Cultural and religious expectations were found to both motivate and complicate caregiving. Interventions such as caregiver education, formal policy support, and the integration of domestic workers were highlighted as potential avenues for relief.

**Conclusion:**

Informal caregivers in the MENA region face a multifaceted burden with limited structural support. Culturally sensitive interventions are necessary to alleviate the psychological, financial, and emotional strain experienced by these individuals, with a focus on education, policy reform, and the development of an inclusive caregiving infrastructure.

## Introduction

Dementia is a complex neurodegenerative disease that affects multiple cognitive domains, including memory, communication, language, attention, reasoning, and visual perception. The global prevalence of dementia has increased at an alarming rate, with over 55 million people worldwide living with the disease (1). This figure is predicted to triple by 2050 due to the aging population (1). This rapid rise in dementia cases contributes to the growing burden on caregivers, who often face physical, emotional, and financial challenges in caring for individuals with dementia (2, 3).

**Figure 1:**
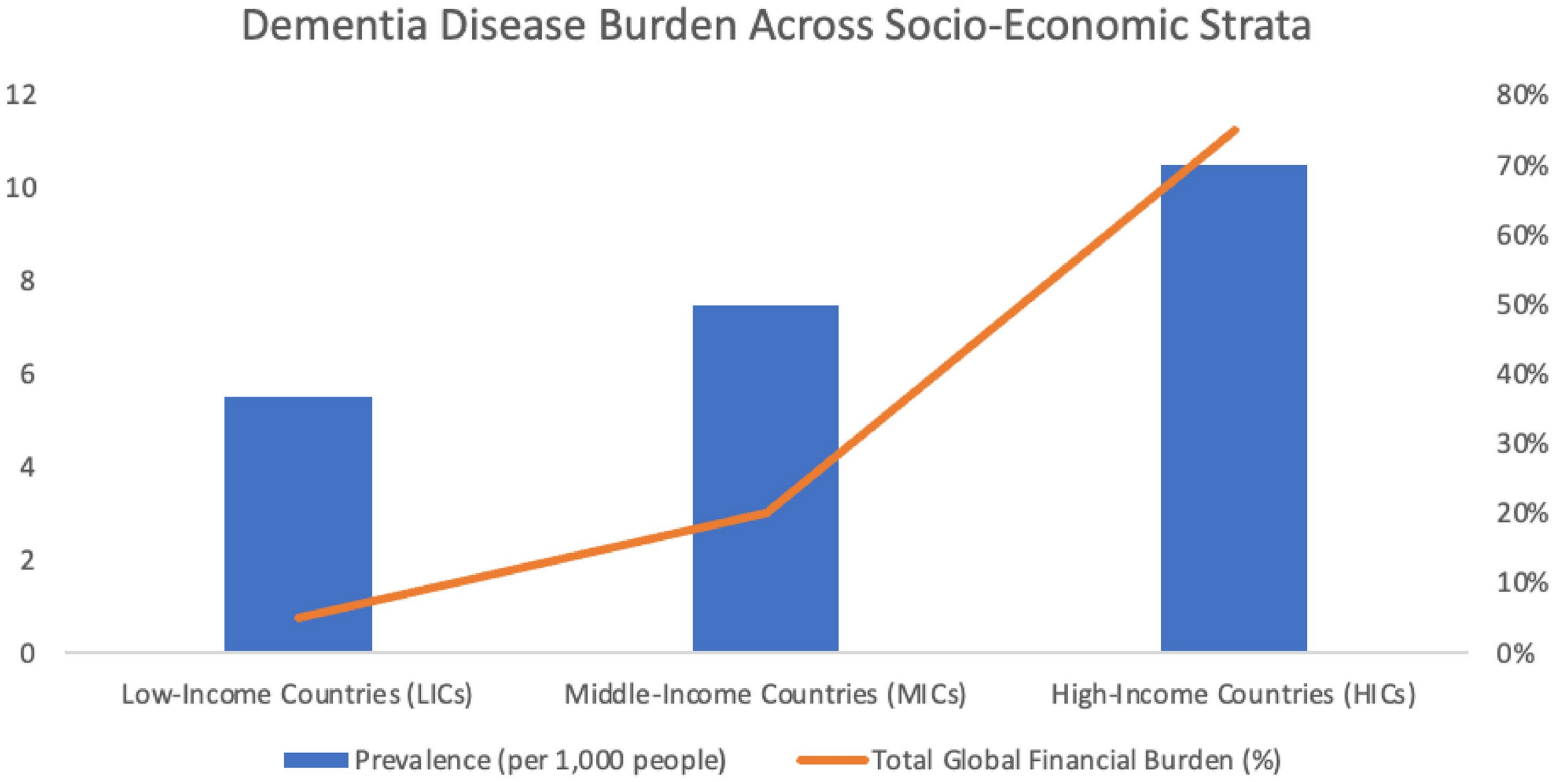
The burden of dementia across low-income countries (LICs), middle-income countries (MICs), and high-income countries (HICs). The left y-axis indicates the prevalence of dementia per 100 people; the right y-axis indicates the total global financial burden (%).

The economic burden of dementia is substantial, with informal caregiving accounting for approximately 50% of the total global financial impact associated with dementia care (4) (Figure 1). In the United States alone, caregivers provide an estimated 18.6 billion hours of unpaid care annually, valued at $244 billion (3).

**Figure 2:**
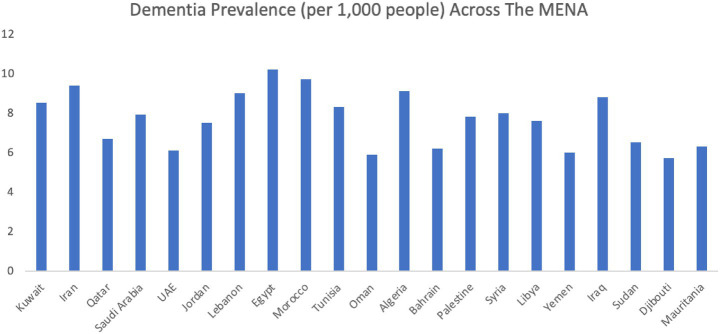
Prevalence of dementia across countries in the MENA region.

While dementia poses challenges for caregivers globally, the burden is especially pronounced in regions with limited formal care infrastructure. In the Middle East and North Africa (MENA) region, cultural expectations significantly shape caregiving practices, imposing immense pressure on informal caregivers (5). Within Arab culture, there is a strong expectation for family members to care for sick relatives, often without adequate support from the state or healthcare infrastructures. This expectation, combined with the scarcity of specialized dementia facilities, places a significant emotional, physical and financial burden on caregivers, who are often left without formal support structures. Research has underscored the growing public health concern of dementia in the Arab world, with an estimated 1,329,729 individuals affected in 2021 and total costs ranging between $10.43 billion and $13.90 billion (6) (Figure 2). The prevalence is exceptionally high in countries such as Lebanon, Tunisia, and Algeria, where dementia rates among individuals over 60 years are among the highest in the MENA region, further exacerbating the challenges faced by carers (6). These statistics underscore the urgent need for enhanced dementia care infrastructure and support for caregivers in the MENA region, as the issue continues to escalate.

In this paper, we examine caregiver burden in the MENA region, focusing on identifying key themes, demographic variations, and the impact of cultural and religious factors. We describe how caregiver burden affects health outcomes, highlight recurring gaps in the literature, and summarize differences in how it has been measured across studies. Our goal is to provide a narrative synthesis of key themes that informs supportive interventions addressing the diverse needs of caregivers. Additionally, based on the reviewed literature, we propose recommendations for alleviating caregiver burden and bridging the gap between research findings and practical application in policy development and community support.

## Methods

This paper presents a narrative literature review of studies exploring the challenges and burdens faced by informal caregivers of individuals with dementia in the Middle East and North Africa (MENA) region. A comprehensive literature search was conducted in January 2024 using five electronic databases: PubMed, Medline, Embase, Scopus, and Web of Science. To ensure broad inclusion, no restrictions were applied regarding language, publication type, or publication date.

Search terms combined controlled vocabulary and free-text keywords, including “caregiver burden,” “informal caregiving,” “dementia,” “family caregiver,” “Alzheimer’s,” “MENA,” and the names of individual MENA countries (e.g., Egypt, Lebanon, Saudi Arabia). Boolean operators were used to connect terms. Studies were included based on their relevance to the research question and their contribution to understanding the experiences, challenges, and needs of informal caregivers within MENA populations. A total of 32 studies met the inclusion criteria and were reviewed.

**Figure 3:**
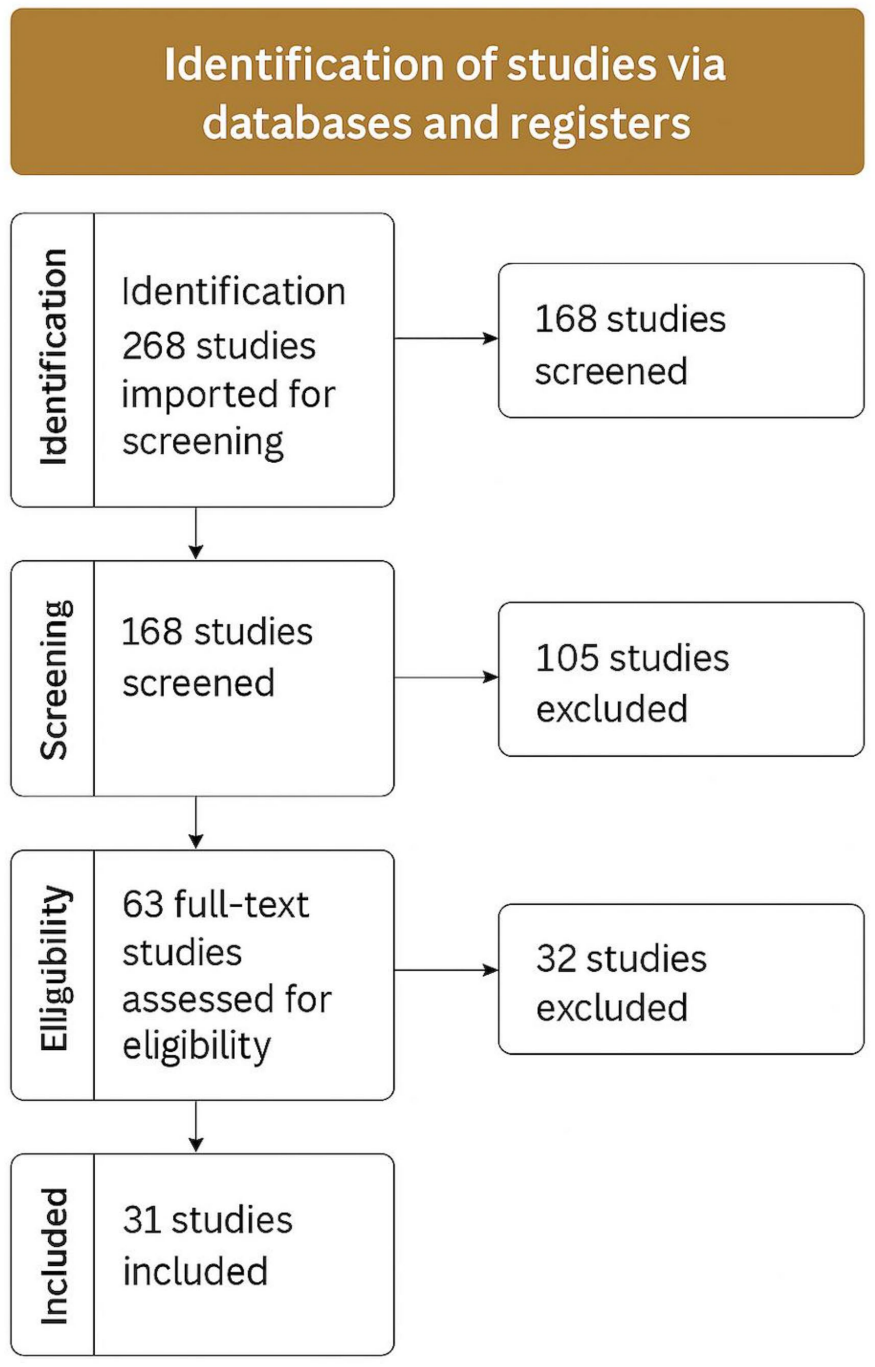
PRISMA 2020 flow diagram illustrating the study selection process for the systematic review. A total of 268 records were identified through database searches. After removing 100 duplicates, 168 records were screened, and 63 full-text articles were assessed for eligibility. Ultimately, 31 studies met the inclusion criteria and were included in the final review.

**Figure 4:**
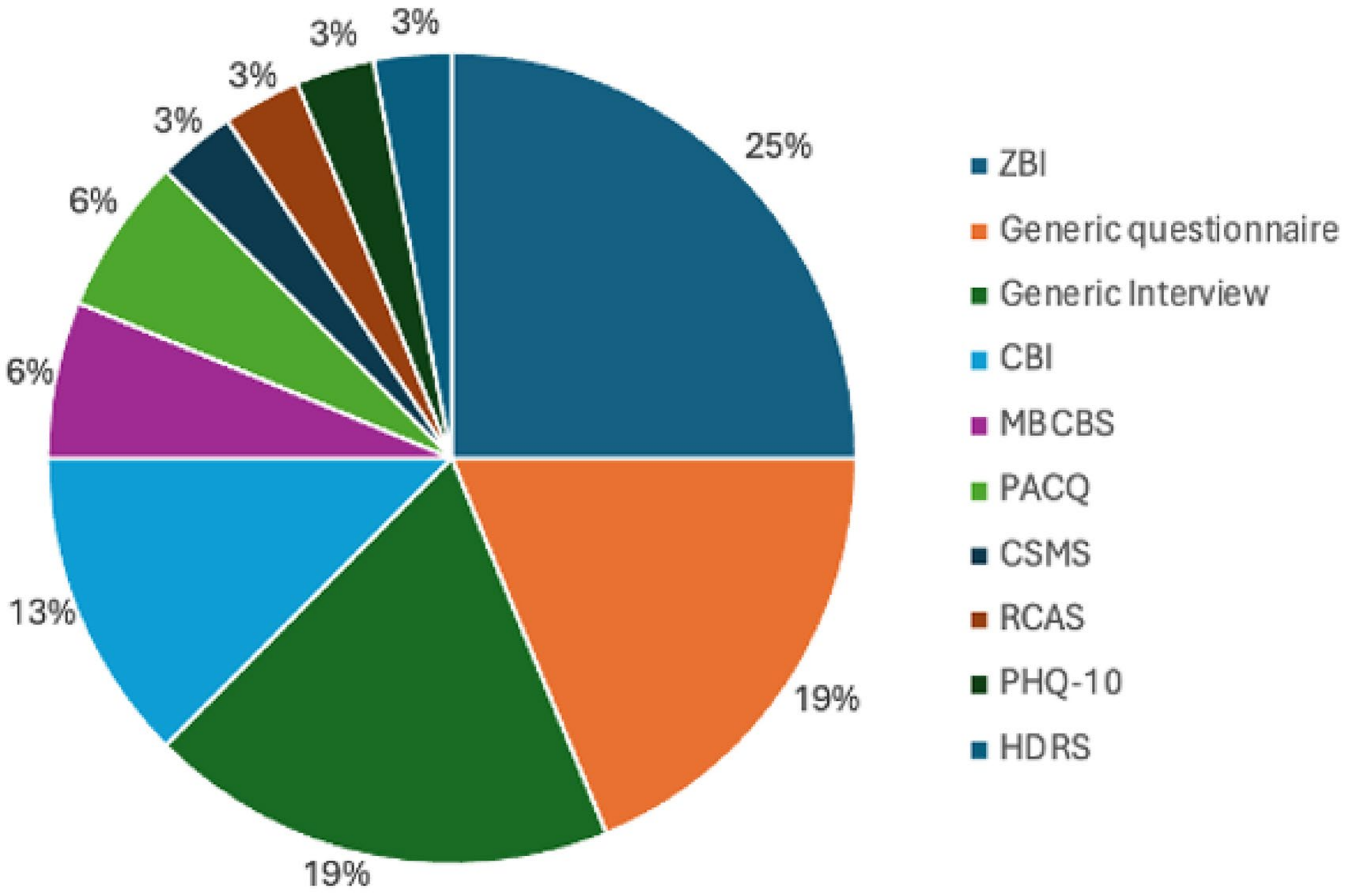
Instruments used to measure caregiver burden. ZBI, Zarit Burden Interview; CBI, Caregiver Burden Inventory; MBCBS, Montgomery-Borgatta Caregiver Burden Scale; PACQ: Patient Assessment Caregiver Questionnaire; CSMS: Caregiver Strain Measure Scale; RCAS: Revised Caregiver Appraisal Scale; PHQ-10: Patient Health Questionnaire (10-item version); HDRS: Hamilton Depression Rating Scale.

The included studies employed a variety of research designs, including cross-sectional surveys, qualitative interviews, and mixed-methods approaches. Measurement tools for caregiver burden varied; the Zarit Burden Interview (ZBI) was the most frequently used, followed by the Caregiver Burden Inventory (CBI) and other regionally adapted instruments. The distribution of these tools is illustrated in Figure 3, reflecting their cultural appropriateness and perceived validity across diverse Middle East and North Africa (MENA) contexts (Figure 4).

We informally drew upon the Pearlin Stress Process Model to guide our thematic synthesis, allowing for the contextualization of caregiver burden across emotional, financial, and social domains. Data were extracted and organized using inductive thematic analysis. Key themes—such as caregiver demographics, psychological strain, economic challenges, access to support services, and the influence of cultural and religious norms—were identified across studies. Two reviewers independently categorized findings, and any discrepancies were resolved through consensus to enhance consistency.

Given the diversity of study designs, populations, and outcome measures, we did not conduct a meta-analysis. Furthermore, as this work constitutes a narrative (rather than systematic) review, it was not registered in PROSPERO, nor was a formal risk of bias tool applied. Instead, we prioritized thematic depth, cultural insight, and conceptual breadth, providing a comprehensive regional synthesis of informal dementia caregiving.

## Results

### Demographic characteristics of caregivers

In the MENA region, studies consistently report that informal caregiving for individuals with Alzheimer’s disease and related dementias is primarily provided by women in their 40s who are stay-at-home parents, as well as by children. These caregivers typically dedicate over 4 h a day to patient care (6, 7). Additionally, most of these caregivers are married and live in the same household as the patient, although there is a significant difference in the level of educational attainment among them, highlighting the substantial demographic variations present in the caregiving burden (8).

Caregivers encounter distinct challenges due to their emotional closeness to the patient, particularly spouses and children, who constitute the majority of caregivers. This emotional proximity can intensify the perceived burden, as highlighted in studies that emphasize the emotional toll on spouses (9). The financial impact on caregivers is considerable, as many also assume the economic responsibilities of care. Caregivers with minimal informal social support, restricted financial resources, and longer hours devoted to care are more likely to experience significant burdens (10).

### Economic burden

The economic burden of caregiving in the MENA region is worsened by a lack of governmental support and the high costs associated with dementia care. Many caregivers experience significant financial strain, with some compelled to reduce their work hours, deplete savings, or modify their professional lives to manage caregiving expenses (9). Some studies indicate that up to 69.1% of Alzheimer’s patients have a monthly income insufficient to meet their daily needs, leaving caregivers to shoulder the financial responsibilities of healthcare, medications, and daily living costs, further intensifying their financial pressures (11).

The shortage of reliable dementia care facilities in the MENA region further exacerbates the burden on family members, who are often left to take on additional responsibilities. Studies commonly highlight that caregivers frequently assume the responsibility of managing patients’ healthcare, medications, and everyday living expenses, which places even greater financial demands on them. This issue is particularly concerning as governmental aid is often insufficient or absent, leaving caregivers to manage these challenges with minimal external support (12).

### Patient characteristics and caregiver burden

The literature suggests that caregiver burden is influenced by several patient-related factors, such as age, place of residence, chronic health conditions, and the duration of depression (13). Studies indicate that carers supporting patients in the later stages of dementia often experience higher levels of stress, physical exhaustion, and emotional strain (14). This is particularly true when caregiving responsibilities are prolonged, as the cumulative effects of managing complex health needs can become overwhelming. In the MENA region, studies report that the typical dementia patient is often male, over 70 years old, and has been living with the disease for more than three years (15). This demographic profile further highlights the challenges faced by carers, as older patients with longer disease durations and additional health issues often require more intensive care, thus increasing the demands placed on their helpers. As a result, caregivers in these situations are more prone to experiencing heightened physical and emotional burdens over time.

The various types of Alzheimer’s disease and related dementias (ADRD) present a range of unique challenges for caregivers, many of whom often lack the specialized knowledge and experience needed to manage the associated comorbidities effectively. For instance, recognizing symptoms such as cognitive decline, understanding the increased risk of infectious diseases, and managing behavioral disturbances are essential skills that caregivers must develop to address these comorbidities effectively (8). Additionally, whether the illness progresses steadily, slowly, or rapidly, it significantly shapes the caregivers’ long-term plans, emotional well-being, and expertise. A slow decline may allow for gradual adaptation, while a rapid progression can overwhelm caregivers emotionally and logistically. Over time, caregivers must continually adapt to the changing needs of the patient, which can place a considerable strain on their well-being and require ongoing learning to enhance their caregiving expertise.

### Governmental aid and financial responsibility

The reviewed literature frequently emphasizes the inadequacy of government support for caregivers in the MENA region. In several countries, secure government facilities for seniors are scarce, placing an additional financial burden on caregivers. For example, 90.3% of caregivers in Egypt report inadequate income (12, 14). This reported lack of support contributes to the stress faced by caregivers, leading to increased levels of anxiety and depression reported by them.

Additionally, national strategies tailored to each country’s unique culture and demographics are crucial for effectively addressing dementia. Recognizing this, the WHO Global Strategy for Dementia has called for governments to implement strategies by 2025 that raise public awareness and improve health, social care, and support for people with dementia and their families (2). Countries such as Qatar, Iran, and Kuwait in the Middle East and North Africa (MENA) region have begun to develop these strategies, with other nations in the area following suit (Table 1). Despite initial steps in some countries, government support in the MENA region remains widely reported as insufficient, with very few caregivers receiving financial support, leaving many to meet the economic challenges of caregiving alone. This situation highlights the urgent need for more comprehensive policies and resources to support caregivers, ensuring they are not faced with these challenges without the help they desperately need.

**Table 1:** Summary of national strategies and support programs for dementia caregivers in selected MENA countries.

MENA country	National strategy	Aims and objectives	Specific services provided	Target populations	Funding sources	Policy impact	Reference
Qatar	Qatar National Dementia Plan	Respite care. Support groups. Education programs.	Respite care: in-home and community-based options for caregivers. Education programs for caregivers, including skill-building in stress management and dementia care.	Family caregivers in urban and rural areas.	Public sector funding from the Ministry of Public Health. Partnerships with local healthcare organizations.	Advantages: Increased engagement of caregivers in support groups. Improved caregiver well-being and knowledge. Limitations: Limited outreach in rural areas.	(30)
Iran	Iranian Alzheimer’s Association Caregiver Program	Regular support group meetings. Educational workshops for caregivers. Resources for caregivers (e.g., guides, videos).	Weekly support group meetings led by healthcare professionals. Workshops on specific aspects of dementia care. Distribution of digital and physical resources.	Caregivers in urban centers, primarily family members caring for the elderly with moderate-to-advanced dementia.	Iranian Alzheimer’s Association. Partial government support.	Advantages: Greater access to caregiver training materials. Limitations: Lack of resources for rural caregivers. Cultural challenges in sharing caregiving responsibilities.	(31)
Kuwait	Kuwait Alzheimer’s Society	Raise awareness of dementia. Support caregivers with respite options.	Awareness campaigns through TV, radio, and social media. Limited formal respite care options through partnerships with local healthcare providers.	Urban population, primarily spouses and children caring for elderly family members.	Kuwait Ministry of Health and private donations.	Advantages: Increased public awareness. Limitations: Gaps in formal caregiving support infrastructure.	(18)
UAE	Memory clinic	Comprehensive training workshops for caregivers. Respite care options.	Monthly workshops covering dementia symptoms, caregiving strategies, and stress relief techniques. Respite care options provided through healthcare institutions.	Focused on caregivers in urban areas, predominantly family members.	Partially funded by the Ministry of Health. Collaboration with private clinics and international dementia organizations.	Advantages: High engagement in urban centers. Limitations: Need for expansion of services to rural areas and marginalized populations	(31)
Lebanon	IDRAAC	Workshops for caregivers. Mental health counseling for caregivers. Resource guides for managing dementia.	Bi-monthly workshops on mental health, dementia care strategies, and self-care for caregivers. Access to mental health counseling. Distribution of printed and online resource guides.	Primarily urban caregivers. Mental health support targeted at caregivers experiencing burnout.	Supported by non-governmental organizations (NGOs), with partial international funding.	Advantages: Improved caregiver mental health outcomes Limitations: Challenges with accessibility in rural areas due to political and economic instability.	(32)
Egypt	Cairo Alzheimer’s Association	Training programs for caregivers. Support groups. Educational resources.	Comprehensive caregiver training programs on managing dementia symptoms and communication. Support groups led by trained facilitators Distribution of digital educational content.	Primarily focused on caregivers in Cairo and other major cities Some outreach to rural areas through mobile clinics.	Ministry of Health funding and international dementia research grants.	Advantages: Improved caregiver skills and confidence Limitations: Limited access to programs in rural areas	(32)
Saudi Arabia	National Transformation Program	Provide training programs for caregivers. Offer support groups. Develop educational resources on dementia.	Regularly scheduled workshops in major cities on dementia management. Online support groups to address geographical challenges. Educational resources via printed and online formats.	Caregivers in urban areas. Limited outreach in rural areas.	Saudi Ministry of Health. Collaborations with global organizations for dementia care expertise.	Advantages: Significant improvements in caregiver knowledge and engagement. Limitations: High demand for expansion to rural areas.	(32)

The table outlines key initiatives, services, funding sources, target populations, and reported policy impacts.

### Challenges faced by informal caregivers

Informal caregiving for individuals with dementia often falls on women, who experience significantly higher levels of stress, physical burden, and emotional fatigue. The age and gender of caregivers influence this burden, with informal female caregivers, particularly housewives, reporting the greatest strain due to the complex demands of caregiving (12). Several studies identify the “sandwich generation” phenomenon as a key contributor to this increased burden, where many women caring for dementia patients are also responsible for their children and aging parents. This dual responsibility often leads to emotional and physical fatigue, compromising their ability to provide adequate care for both their family and the dementia patient. As a result, their families’ quality of life can suffer (12). Women also tend to take on more hands-on and complex caregiving tasks. Unmet needs and stress often accompany these heightened demands, as women are less likely to utilize formal and informal support networks compared to their male counterparts (16). This lack of support further compounds the challenges they face, making it even more difficult for them to cope with the multiple demands of caregiving.

### Knowledge gaps

A recurring theme in the literature is that caregivers in the MENA region face the challenge of lacking knowledge about dementia and how to manage it. Informal caregivers often have a limited understanding of the disease, which can lead to misjudgments about the capabilities of people with dementia (PWD). This misunderstanding can create unsafe conditions for the patient, as caregivers may not fully recognize the extent of the patient’s impairments. Some studies report that between 25 and 50% of caregivers may overestimate the abilities of people with disabilities (PWD) to perform basic tasks, such as telling time, managing finances, and maintaining personal hygiene, even when those skills have significantly declined (17). The absence of suitable educational and support systems within the healthcare infrastructure further exacerbates the knowledge gap for caregivers. A recent study in Kuwait found that among healthcare professionals, only 10.4% of participants reported attending continuing professional development (CPD) courses or conferences on dementia (18), underscoring the broader lack of focus on dementia education, even among professionals.

Addressing these gaps necessitates improved education and access to resources. Enhanced awareness and training would elevate the quality of patient care while alleviating the stress faced by caregivers. National policymakers play a critical role in raising public awareness of dementia and developing comprehensive, evidence-based resources to support caregivers. Initiatives such as the Global Dementia Observatory (GDO) exemplify how governments can furnish care with the necessary information and support to manage the challenges of dementia care more effectively (18).

### Impact on marital status and careers

Caregiving responsibilities often place significant strain on marital relationships and careers, particularly for women who balance caregiving duties with professional obligations. Some studies suggest that the stress from caregiving may be especially burdensome for spouses, who often report a higher burden than other family members (19). This increased burden is attributed to the extended periods of caregiving that spouses typically provide, which heightens the risk of both physical and mental health issues commonly associated with caregiver strain (2).

The literature presents mixed findings regarding the impact of marital status on caregiver stress. While some evidence suggests no significant difference in stress levels between single and married caregivers (12), other findings indicate that spouses experience a higher burden (12). Employment status is also reported inconsistently across studies when examining caregiver stress. While employment status appears to have little effect on depression levels, unemployed caregivers (8), particularly those financially responsible for the patient, are at a greater risk of experiencing depression (9).

Employed caregivers often confront role conflicts as they balance their professional and caregiving responsibilities. This conflict can lead to workplace challenges, such as needing to take multiple days off, arriving late, or leaving early. Consequently, many caregivers find themselves at a heightened risk of job termination or reduced income, further compounding their stress and financial difficulties (20).

### Increasing demands and economic challenges for working caregivers

Competing demands and economic challenges significantly exacerbate the burdens faced by working caregivers, as indicated in studies that examine the intersection between caregiving responsibilities and financial pressures (8). Nearly half of all caregivers are employed alongside their caregiving duties, compelling them to navigate the delicate balance between workplace commitments and caregiving needs. This dual role stretches their physical and emotional capacities, placing them in a precarious financial situation.

Economic challenges exacerbate the overall burden on caregivers, with financial strain often contributing to the development of depressive symptoms (8). This correlation stems from increased financial strain that intensifies the overall burden on caregivers and underscores the direct impact of economic challenges on their mental health. Similarly, a study by Andrén and Elmståhl (21) investigated the characteristics of family caregivers, revealing that low income coupled with poor subjective health, particularly among adult caregivers, is associated with an elevated caregiver burden. These individuals frequently encounter more significant challenges than other family caregiver groups, irrespective of age, highlighting the need for targeted interventions to support them.

### Cultural and religious influences on caregiving

Cultural norms in the MENA region are often described as significant influences on caregiving roles, strongly emphasizing familial duties and honor (22). These traditions typically place the caregiving burden primarily on family members, with women shouldering most of the responsibility, even when they lack adequate support systems to alleviate these challenges (22). In many cases, caregiving is regarded as an intrinsic part of a woman’s role, and this cultural expectation often results in the under-recognition of their contributions (12).

In Lebanese society, traditional values prepare women for lifelong caregiving roles that encompass looking after children, parents, and siblings while also managing their personal lives. This stands in contrast to the upbringing of boys, who are encouraged to develop independence and emotional resilience, thereby reinforcing gender disparities in caregiving responsibilities. Similarly, cultural beliefs regarding old age, health, and mental health can shape perceptions of Alzheimer’s Disease (AD), potentially leading to stigma and discrimination against those affected by the condition and their caregivers (16).

Religious beliefs in the MENA region can significantly influence caregiving practices, often aligning with cultural norms to reinforce the perception that caregiving is primarily a family-based responsibility. Caregiving is frequently viewed as a religious obligation, offering spiritual motivation and a path to divine reward. This intertwining of religious duty with caregiving tasks often requires more formal support, as caregivers may hesitate to seek help due to religious or cultural expectations (23).

The MENA region is predominantly Muslim, and Islamic caregiving principles rooted in the Quran emphasize familial obligations and respect for parents. Similarly, religious teachings across Christianity and Judaism also highlight the importance of caregiving within the family, reinforcing cultural norms that assign this responsibility to family members. These teachings are often cited as guiding caregiving behaviors, embedding the practice within the region’s cultural fabric (23). However, while these religious and cultural imperatives provide spiritual motivation, they also underscore the need for greater formal recognition and support for caregivers, thereby further complicating their role.

### The role of domestic workers in caregiving

Domestic workers are increasingly acknowledged in the literature as playing a significant role in caregiving in the MENA region, often undertaking tasks typically performed by family members (24). However, their contributions are frequently overlooked in research and policy discussions, highlighting the need for a deeper understanding and greater support for their role within the caregiving ecosystem. One study from Kuwait indicated that older adults were 10.5 times more likely to rely on a domestic worker for care in the absence of traditional familial caregivers. However, people living with dementia who are cared for by domestic workers report worse health outcomes, including higher levels of disability and depression (24). This observed gap in the literature emphasizes the need for more evidence regarding domestic workers’ roles in caregiving and underlines the necessity for further research on their contributions. Addressing this gap is crucial for enhancing the caregiving experience for individuals with dementia and their families by ensuring a well-supported, trained, and integrated caregiving team (23).

## Discussion

This narrative review highlights the intricate and multidimensional nature of informal dementia caregiving in the MENA region, revealing a complex interplay between demographic, economic, cultural, and structural factors. Drawing on the Pearlin Stress Process Model, the evidence underscores how chronic exposure to emotional and financial stressors—combined with limited coping resources—intensifies caregiver burden over time. These findings mirror global trends in caregiver distress, yet are shaped in distinctive ways by the region’s sociocultural and religious fabric.

### Structural and policy-level gaps

Governmental support for caregivers across MENA remains largely inadequate. Despite the WHO’s call for all countries to implement national dementia strategies by 2025 (2), only a handful, such as Qatar and Iran, have made notable progress; even these initiatives face significant barriers regarding rural outreach, funding, and sustainability. The vast majority of caregivers operate in contexts with limited formal services, such as respite care, community-based training, or home support. This policy vacuum leaves informal caregivers, particularly women, bearing the brunt of emotional and financial responsibilities (12, 14).

Domestic workers are increasingly filling gaps in care, yet their roles remain underregulated and underappreciated. As studies show, households that rely heavily on domestic workers, often without appropriate training or oversight, report worse health outcomes for dementia patients (24). The absence of legal protections, access to training, or inclusion in social security systems for domestic workers across most MENA countries raises ethical concerns and jeopardizes the quality of care (25). International research similarly affirms that unregulated reliance on migrant caregivers leads to fragmented care, occupational exploitation, and inconsistent outcomes (13, 26).

### Healthcare systems and cultural mediation

Healthcare professionals in the MENA region play a pivotal yet underutilized role in dementia care. Most receive minimal training in geriatric mental health, leaving caregivers unsupported when navigating dementia-related challenges (18). This deficit is particularly pronounced in primary care settings, where providers are often the first point of contact but lack specialized resources.

However, effective intervention requires more than medical knowledge; it demands cultural competence. Interventions that disregard the central role of religion, family hierarchy, and gender expectations may be perceived as intrusive or irrelevant. In predominantly Muslim societies, caregiving is not simply a responsibility but a sacred act. Religious teachings emphasizing care for parents can both empower and constrain caregivers, particularly when they feel guilty seeking help (22, 23). Therefore, healthcare workers must blend clinical support with cultural sensitivity, reinforcing caregivers’ strengths while gently addressing any knowledge or practice gaps.

### Education and intervention strategies

Educational interventions show promise in alleviating caregiver burden. The Progressively Lowered Stress Threshold (PLST) model has demonstrated its ability to reduce caregiver distress by promoting strategies that align with dementia patients’ fluctuating stress tolerance levels (27). Meanwhile, the WHO’s iSupport booklet, adapted for use in Tunisia, provides a scalable self-help framework offering psychosocial guidance and practical care tips (28).

Yet, broader access and contextual adaptation remain essential. Several studies have revealed that caregivers who receive information from trained professionals report a lower perceived burden than those relying on informal channels, such as social media (29). This points to an urgent need for trusted, evidence-based information pathways—ideally led by culturally literate health professionals or trained community workers. Government and NGO collaboration could support mobile apps, printed guides, and religiously framed workshops to improve uptake across diverse literacy and income levels.

### Gender and the invisible labor of women

Gender dynamics emerged as a powerful and persistent theme. Across the MENA region, caregiving is overwhelmingly feminized, with women—often housewives or members of the “sandwich generation”—providing care while managing household and child-rearing duties (12, 16). This dual role usually leads to emotional exhaustion, social isolation, and economic vulnerability. While similar patterns exist globally, the cultural intensity of gendered caregiving expectations in MENA renders these burdens more entrenched.

Addressing this requires gender-sensitive policy design. Subsidies, tax relief, caregiver pensions, and legal employment protections for women caregivers could provide long-overdue recognition and relief. Without such measures, the cost of caregiving will continue to be borne by individual women, with implications for mental health, workforce participation, and family wellbeing.

### Future research and regional strategy

To strengthen evidence-based policy and practice, future research should focus on longitudinal studies that assess the long-term physical and psychological impacts of caregiving. Comparative studies across MENA sub-regions can illuminate context-specific challenges and strengths. Equally important are trials evaluating the effectiveness of culturally and religiously adapted educational programs and caregiver support initiatives. Additionally, the integration of domestic workers into formal care frameworks—through training, legal recognition, and structured oversight—remains an underexplored yet vital frontier.

### Limitations

The scarcity of empirical studies on dementia caregiving in Arab speaking countries constraints this narrative review. Most available research is cross-sectional, small-scale, and lacks longitudinal or community-based designs, limiting generalizability and causal insights. Further, methodological variation, such as different dementia diagnostic criteria, inconsistent caregiver outcome measures, and diverse sociocultural settings, posed challenges for synthesis and comparison across studies. Lastly, publication bias is possible given reliance on English and Arabic language sources, which may miss important local reports or studies that aren’t formally published or widely accessible. Together, these factors highlight the need for larger, longitudinal, and culturally adaptive research to better understand caregiving burdens in the Arab world.

## Conclusion

Caregiving for individuals with Alzheimer’s disease and related dementias in the MENA region presents a complex web of psychological, emotional, and financial burdens. This review highlights the multifaceted nature of caregiver strain, particularly among women who predominantly assume these roles. Caregivers, especially women, have been shown in various studies to experience significant emotional exhaustion and depressive symptoms due to the dual responsibilities of managing household duties and caregiving. This dual burden, compounded by a lack of government support, often results in heightened stress levels and financial instability.

Addressing these challenges requires a holistic approach that includes educational programs to reduce the knowledge gap, policy interventions to provide financial relief, and culturally sensitive support systems that align with the values and obligations of the MENA region. Additionally, recognizing and integrating the role of domestic workers into caregiving strategies can optimize their contributions and alleviate the burden on family caregivers. Implementing these measures may help improve the well-being of caregivers and enhance the quality of life for individuals with dementia, ensuring a more supportive and sustainable caregiving environment throughout the MENA region.

## Data Availability

The original contributions presented in the study are included in the article/supplementary material, further inquiries can be directed to the corresponding author.
